# Supplementary material for: ZFP30 promotes adipogenesis through the KAP1-mediated activation of a retrotransposon-derived Pparg2 enhancer
Source: Nat Commun. 2019 Apr 18;10:1809. doi: 10.1038/s41467-019-09803-9 (PMC6472429; doi:10.1038/s41467-019-09803-9)
Supplement: Supplementary file 1 — Supplementary Information [file 41467_2019_9803_MOESM1_ESM.pdf]

ZFP30 promotes adipogenesis through the KAP1-mediated activation of a retrotransposon-derived *Pparg2* enhancer

Chen et al.

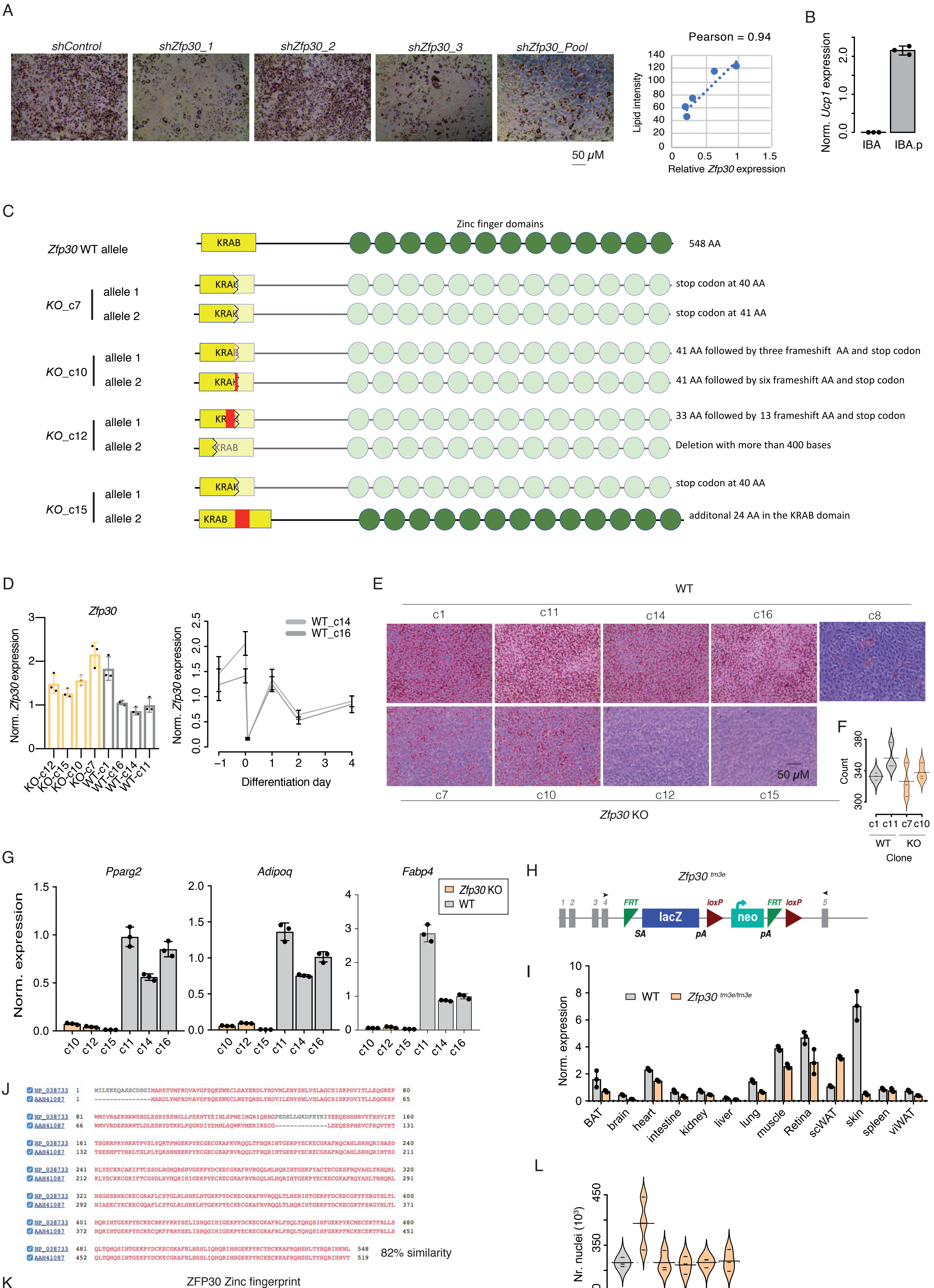

Supplementary Figure 1

Supplementary Figure 1, related to Figure 1: ZFP30 is a positive regulator of adipogenesis

(A) **Left panel:** Microscopic images showing the effect of knocking down *Zfp30* or the negative control (shControl) on 3T3-L1 adipogenic differentiation, as assessed by lipid accumulation (ORO staining); scale bar - 50 µm. Right panel: The ORO staining intensity from Figure 1A was quantified and correlated to relative *Zfp30* mRNA expression. (B) *Ucp1* expression in the parental IBA cells (IBA.p) as well as the IBA subclone (IBA) employed for all experiments presented in the current study. (C) Overview of the CRISPR/Cas9 induced mutation spectrum in the four KO (c7, c10, c12, and c15) clones employed in our study. The red box indicates additional amino acids, jagged lines show premature stop codons. (D) Left panel: *Zfp30* expression using primers away from the CRISPR/Cas9 targeting site. Right panel: *Zfp30* expression during adipogenesis in two distinct WT and KO IBA subclones. (E) Microscopic images showing the effect of knocking out *Zfp30* on IBA adipogenic differentiation as assessed by lipid accumulation (ORO staining); scale bar - 50 µm. (F) Number of nuclei in differentiated *Zfp30* KO and WT IBA cells. (G) Adipogenic marker gene expression in three WT and three *Zfp30* KO IBA subclones post differentiation. (H) Schematic of the ZFP30 targeting cassette in the $Zfp30^{tm3e}$ allele. SA: splicing acceptor, pA: polyA signal. Arrow: the primer for qPCR in (I). (I) *Zfp30* gene expression across distinct mouse tissues in $Zfp30^{tm3e/tm3e}$ and WT mice. BAT - brown adipose tissue, scWAT – subcutaneous white adipose tissue; viWAT – visceral white adipose tissue. (J) Alignment of the human and mouse ZFP30 protein sequence. (K) Alignment of the mouse and human ZFP30 fingerprint. (L) Number of nuclei corresponding to the experiments visualized in **Figure 1G-H**, assessing the effect of *ZFP30* KD and control on primary SVF cells from human lipoaspirate samples. All panels, n=3 biologically independent experiments. ** $p < 0.01$, * $p<0.05$, t-test. Error bars – SD. Source data are provided as a Source Data file.

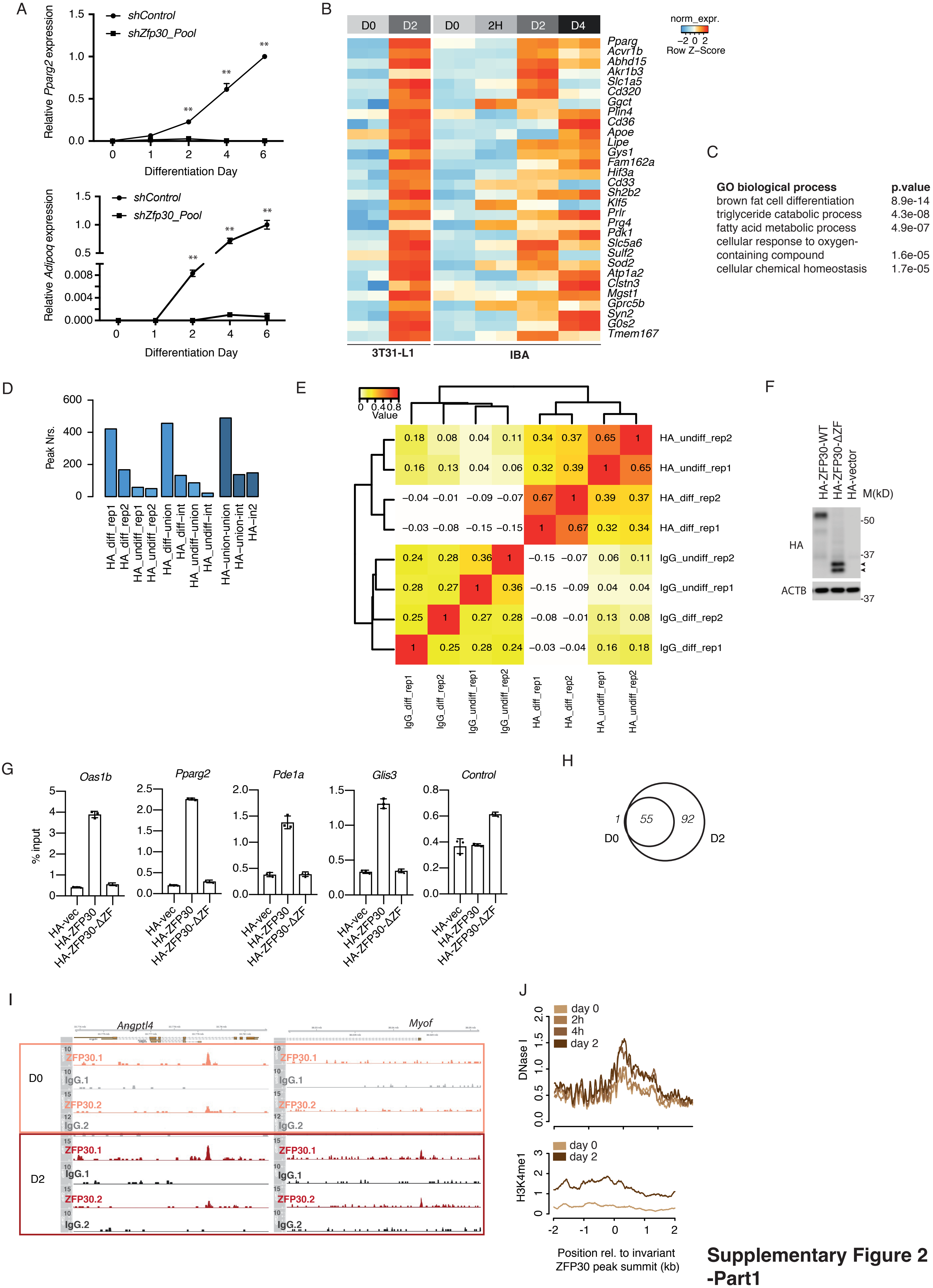

Supplementary Figure 2 -Part1

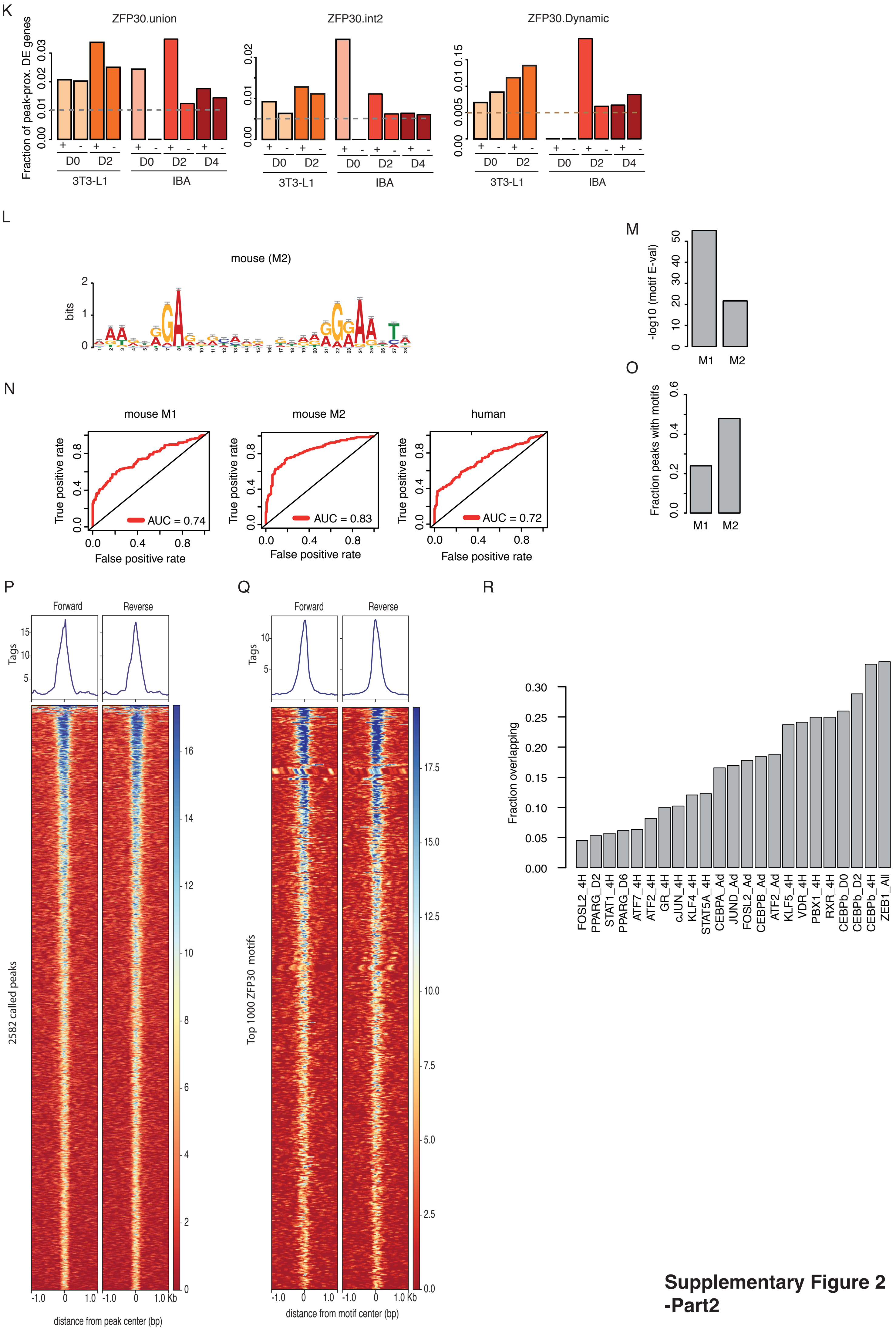

Supplementary Figure 2 -Part2

Supplementary Figure 2, related to Figure 2: ZFP30 regulates multiple targets during adipogenesis

(A) Adipogenic marker (*Pparg* (upper panel) and *Adipoq* (lower panel)) gene expression across six days of 3T3-L1 adipogenic differentiation. n=3 biologically independent experiments. ** $p < 0.01$, t-test. (B) Heatmap displaying the top 30 genes commonly induced upon adipogenic differentiation of the employed 3T3-L1 and IBA cell lines. (C) GO biological processes enriched in genes commonly induced in 3T3-L1 and IBA cells upon adipogenesis. (D) Number of ZFP30 peaks in the individual replicates as well as of the union and intersection of ZFP30 peaks across days. HA-in2: peaks that are overlapping in at least 2 samples, irrespective of days. (E) Correlation (Spearman's rho) between ChIP-seq read counts for all HA-ZFP30 and IgG samples inside the union of ZFP30-bound regions across days and replicates. (F-G) ZFP30 and the ZFP30- ΔZF mutant were introduced into *Zfp30* KO IBA cells, and binding of ZFP30 and the ZFP30-ΔZF mutant to distinct genomic loci was assessed by ChIP-qPCR. (F) Expression levels of the reconstituted proteins were assessed by western blotting. Arrows show two bands for ZFP30- ΔZF. (G) Four ZFP30 targets and one control were evaluated. (H) The overlap between ZFP30-HA ChIP-seq peaks at days 0 and 2. Here, only the peaks present in at least two samples were considered, while in **Figure 2H**, the union of all peaks was considered. (I) Examples of ZFP30-HA DNA binding at the *Algptl4* locus (adipogenic-invariant binding) and *Myof* locus (adipogenic-induced binding). (J) DNase I hypersensitivity (upper panel) and H3K4me1 (lower panel) signal upon adipogenic induction in a 4 kb window centred around the point of maximal ZFP30 binding for invariant ZFP30 peaks. (K) The fraction of genes belonging to distinct categories of differentially expressed genes (up/down regulated in KD/KO, at distinct sampling times across adipogenesis), which are bound by ZFP30. The three individual panels correspond to distinct ZFP30 peak types: ZFP30.union – any peak, ZFP30.in2 – peaks replicated at least once, ZFP30.dynamic – day 2-specific peaks. +-: genes up- and down-regulated in ZFP30 KD or KO cells. The mean overlap value for a randomized control is included as the dashed line (background overlap). (L) Second *de novo* motif (M2) discovered in DNA sequences bound by ZFP30 in 3T3-L1 cells. (M) Motif E-values for the motifs M1 (**Figure 2K**) and M2 (**Supplementary Figure 2L**). (N) ROC plots showing the discriminatory power of ZFP30 motifs to distinguish between ZFP30 peaks and random genomic regions. (O) The fraction of ZFP30 peaks with a significant M1 and M2 motif match, respectively ($p < 0.001$, **Methods**). (P) ChIP-seq reads mapping to the forward and reverse strand centered around the point of maximal ZFP30-HA ChIP-exo enrichment. (Q) Motif hits matching the forward and reverse strand centered around the point of maximal ZFP30-HA ChIP-exo enrichment. (R) The fraction of ZFP30 peaks overlapping regions bound by other TFs previously assessed in 3T3-L1 cells. Source data are provided as a Source Data file.

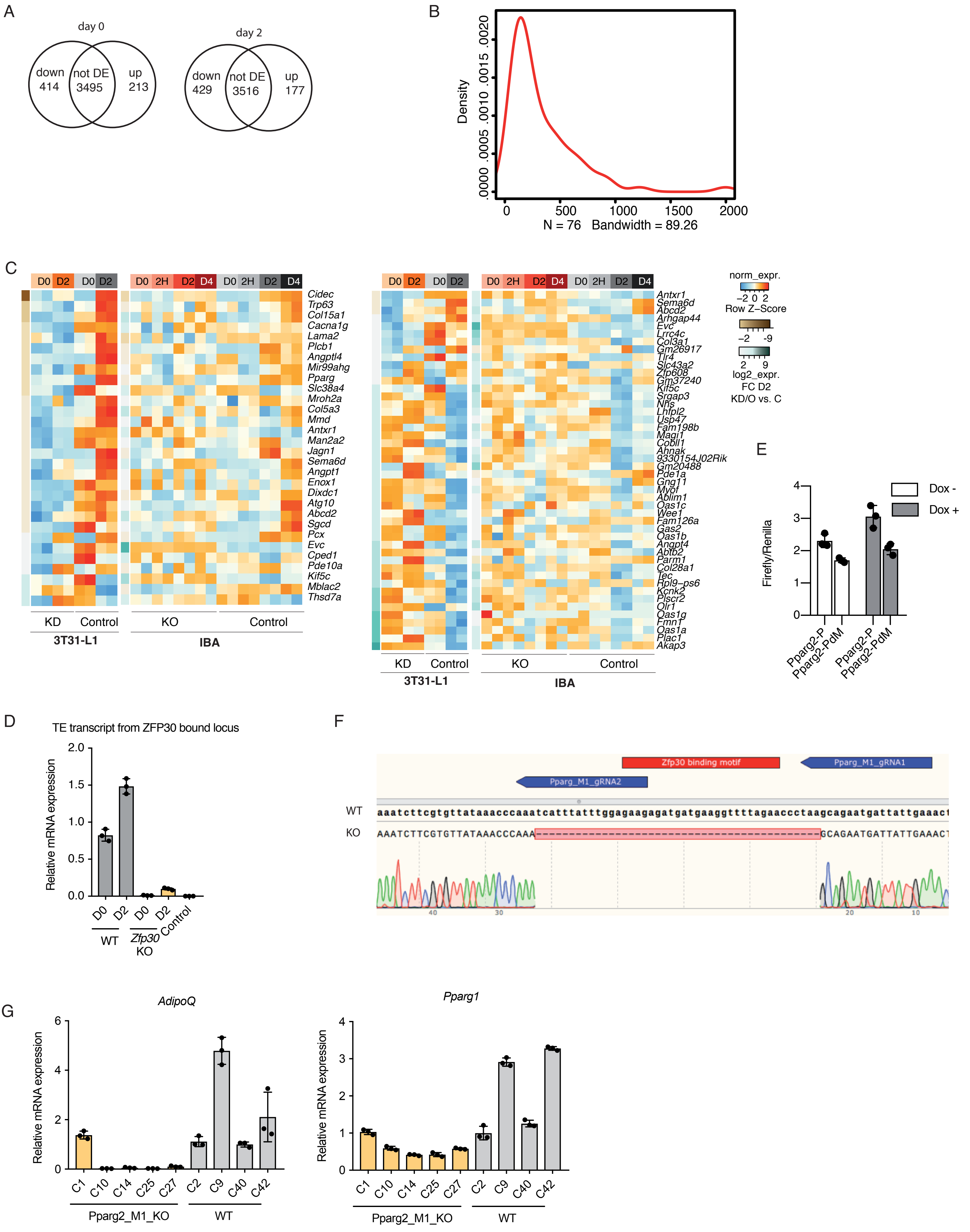

Supplementary Figure 3

Supplementary Figure 3, related to Figure 3: ZFP30 directly enhances *Pparg2* expression during adipogenesis

(A) Venn diagraph showing the number of up- and down- regulated TEs in *Zfp30* KD 3T3-L1 cells. not-DE: not differential expressed. (B) ZFP30-bound L1s tend to be decayed. An intact L1 is typically ~ 6kb. (C) Heatmap displaying all significantly DE genes in KD or KO experiments (down-regulated at any time-point, left; up-regulated at any time-point, right) that also show proximal ZFP30 binding. (D) The transcript derived from the ZFP30-bound TE was assessed by qPCR using random hexamers for reverse transcription in *Zfp30* WT and KO cells. RNA was digested with DNase I before reverse transcription. A non-template control is included. (E) Impact of ZFP30 expression (Dox induced, 0.2 uM) on Pparg2-P and Pparg2-PdM reporter activity. (F) A representative Sanger sequencing result of *Pparg2*-M1 KO cells. (G) The expression of adipogenic genes as assessed by qPCR in *Pparg2*-M1 KO and WT cells. All panels: error bars – SD. Source data are provided as a Source Data file.

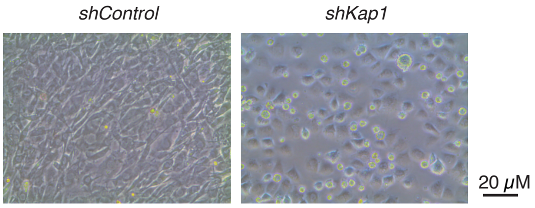

Supplementary Fig. 4

Supplementary Figure 4, related to Figure 4: KAP1 interacts with ZFP30 and promotes adipogenesis

Light/Bright field microscope images of *Kap1* KD and control 3T3-L1 cells.

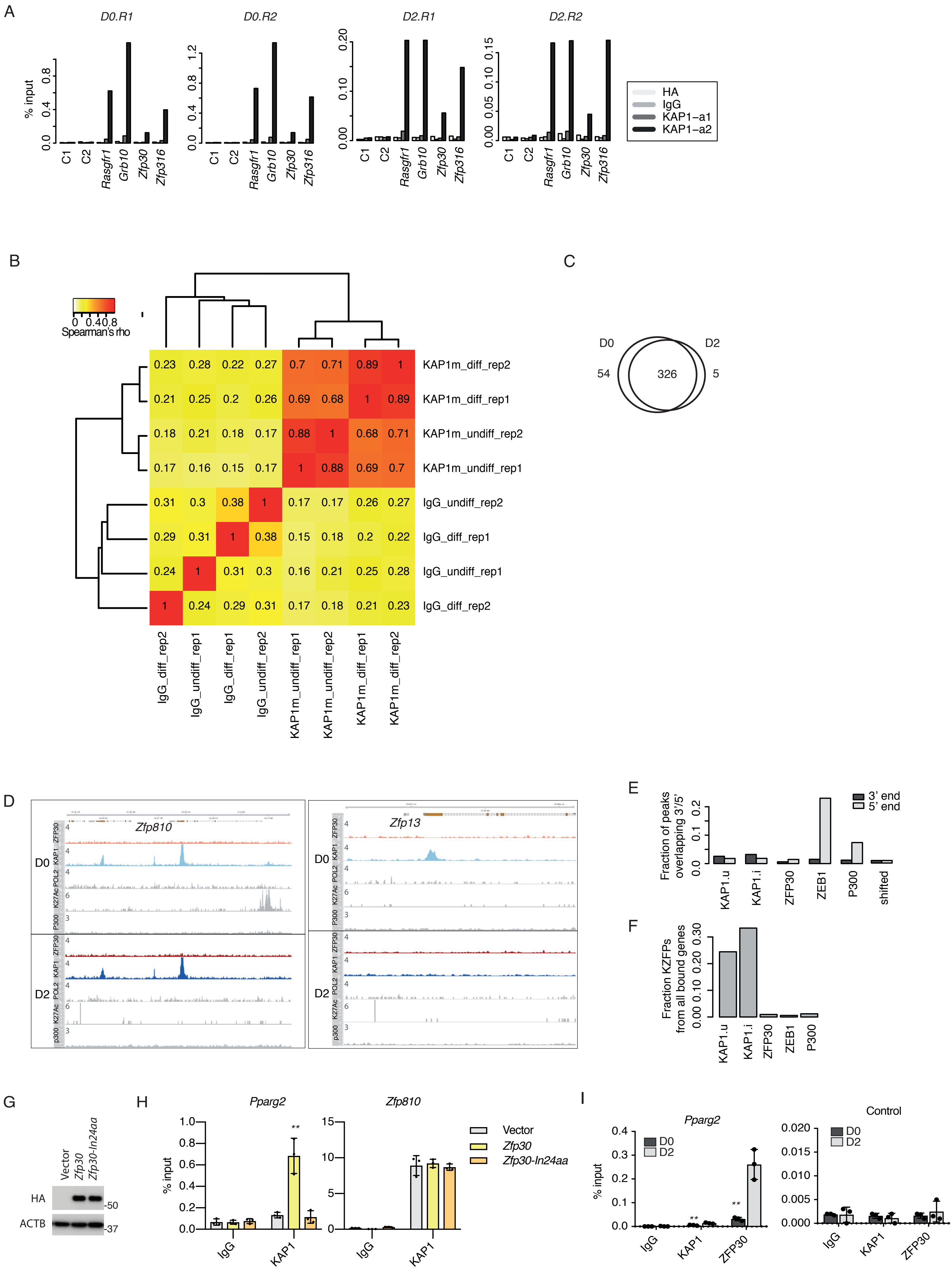

Supplementary Fig. 5

Supplementary Figure 5, related to Figure 5: ZFP30 activates *Pparg2* expression through KAP1

(A) KAP1 ChIP-qPCR of selected loci in 3T3-L1 cells at days 0 and 2 of differentiation. Two different KAP1 antibodies (KAP1-a1: ab10483 (Abcam), KAP1-a2: ab22553 (Abcam)) were used, and IgG was included as a negative control. Four KAP1 binding loci and two negative loci (C1, C2) were tested. Samples from the KAP1-a1 were not used for further processing. (B) Correlation (Spearman's rho) between ChIP-seq read counts for all KAP1 and IgG samples inside the union of KAP1-bound regions across days and replicates. (C) Venn diagram depicting KAP1 ChIP-seq peak overlap between days 0 and 2. Here, only the peaks present in at least two samples were considered, while in **Figure 5B**, the union of all peaks was considered. (D) Examples of KAP1 binding at the *Zfp810* (adipogenic-invariant binding) and *Zfp13* loci (adipogenesis-induced binding), also including ZFP30-HA, RNA Pol II (POL2), p300, and H3K27ac (K27ac) ChIP-seq enrichment tracks at days 0 and 2 of adipogenic differentiation. (E) The fraction of distinct categories of peaks (union of all KAP1 – KAP1.u, KAP1 present in at least two samples – KAP1.i, ZFP30, ZEB1, or p300) overlapping the 3' or 5' end of genes, respectively. (F) The fraction of genes encoding KZFPs from all genes bound by distinct categories of peaks. (G-H) *Zfp30* and the *Zfp30-In24aa* mutant were introduced into *Zfp30* KO IBA cells, and binding of KAP1 to the *Pparg2* enhancer was assessed by ChIP-qPCR. Protein levels are shown in (G), ChIP-qPCR results are shown in (H). n=3 biologically independent experiments. ** p<0.01, t-test. (I) ChIP-qPCR showing that ZFP30 and KAP1 also bind to the same *Pparg2* enhancer locus in IBA cells, similar to that in 3T3-L1 cells. n=3 biologically independent experiments. ** p<0.01, * p<0.05, t-test. All panels: error bars – SD. Source data are provided as a Source Data file.

Fig.1J

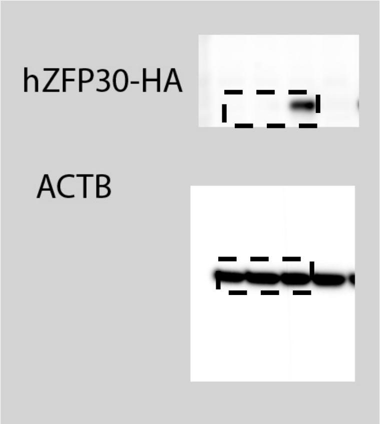

Fig.4A

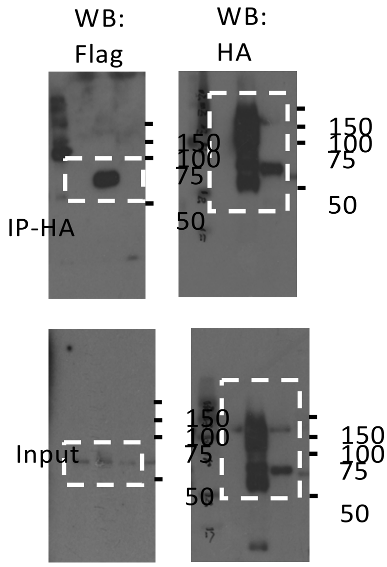

Fig.4B

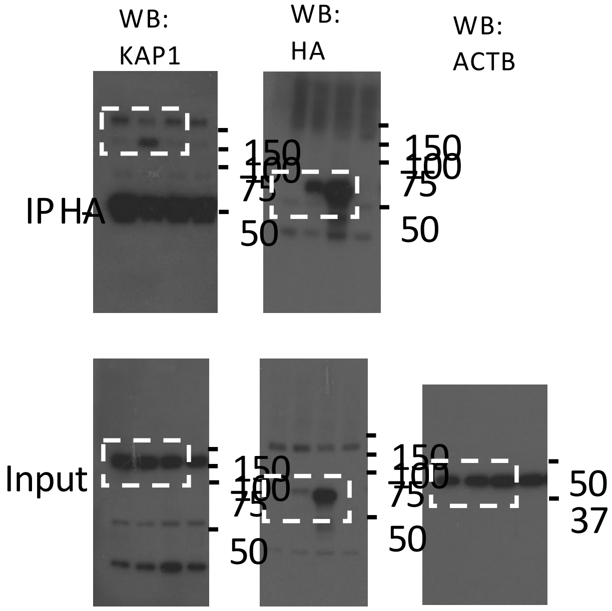

Fig.4C

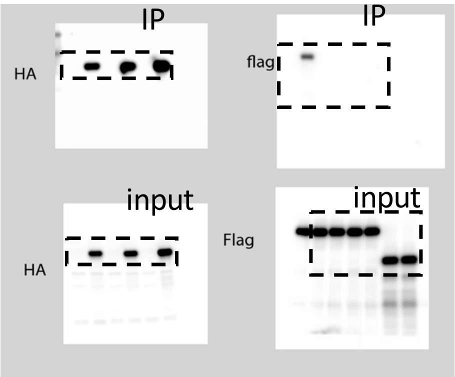

Fig.4E

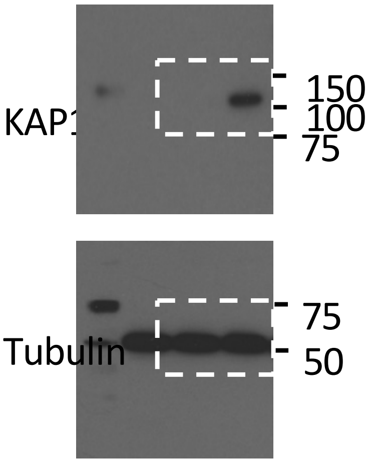

Fig.5G

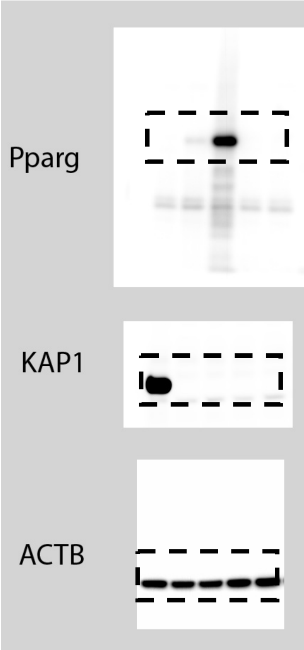

Fig.5H

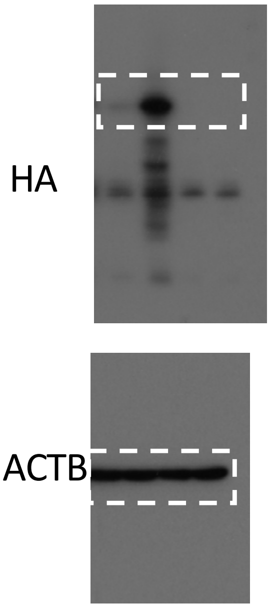

Fig.S2F

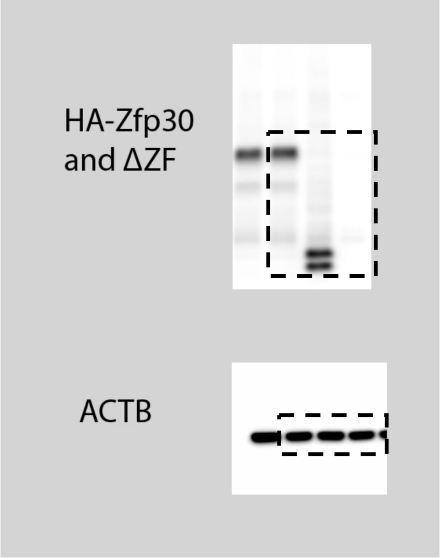

Fig.S5G

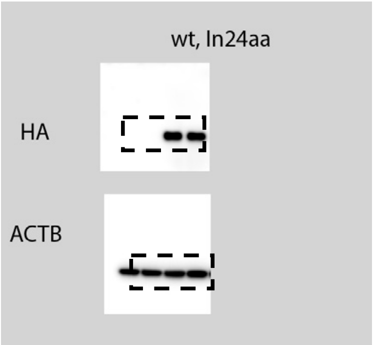

Supplementary Fig. 6

Supplementary Figure 6 Uncropped western blots.
