# Supplementary material for: ZFP30 promotes adipogenesis through the KAP1-mediated activation of a retrotransposon-derived Pparg2 enhancer
Source: Nat Commun. 2019 Apr 18;10:1809. doi: 10.1038/s41467-019-09803-9 (PMC6472429; doi:10.1038/s41467-019-09803-9)
Supplement: Supplementary file 3 — Description of Additional Supplementary Files [file 41467_2019_9803_MOESM3_ESM.pdf]

Description of Additional Supplementary Files

Supplementary Data 1

Description: Genes significantly up- and downregulated upon *Zfp30* KD (3T3-L1) and KO (IBA), respectively

Supplementary Data 2

Description: Gene Ontology biological processes enriched in genes responding to adipogenesis or alterations of *Zfp30* levels

Supplementary Data 3

Description: Correlation between ChIP-seq read counts at ZFP30- and KAP1-bound regions

Supplementary Data 4

Description: Differential ChIP-seq enrichment for ZFP30-HA and KAP1 peaks across adipogenic differentiation

Supplementary Data 5

Description: Known TF binding sites that are significantly enriched in ZFP30-bound regions

Supplementary Data 6

Description: L1ME repetitive elements overlapping ZFP30-HA ChIP-seq peaks

Supplementary Data 7

Description: List of the adipogenic transcription factors used for the motif scanning of the Pparg2_M1 deleted DNA fragment.
