# Supplementary material for: ZFP30 promotes adipogenesis through the KAP1-mediated activation of a retrotransposon-derived Pparg2 enhancer
Source: Nat Commun. 2019 Apr 18;10:1809. doi: 10.1038/s41467-019-09803-9 (PMC6472429; doi:10.1038/s41467-019-09803-9)
Supplement: Supplementary file 11 — Reporting Summary [file 41467_2019_9803_MOESM11_ESM.pdf]

nature research

Corresponding author(s): Bart Deplancke

Last updated by author(s): Feb 28, 2019

Reporting Summary

Nature Research wishes to improve the reproducibility of the work that we publish. This form provides structure for consistency and transparency in reporting. For further information on Nature Research policies, see Authors & Referees and the Editorial Policy Checklist.

Statistics

For all statistical analyses, confirm that the following items are present in the figure legend, table legend, main text, or Methods section.

n/a	Confirmed	
☐	☒	The exact sample size (*n*) for each experimental group/condition, given as a discrete number and unit of measurement
☐	☒	A statement on whether measurements were taken from distinct samples or whether the same sample was measured repeatedly
☐	☒	The statistical test(s) used AND whether they are one- or two-sided<br>*Only common tests should be described solely by name; describe more complex techniques in the Methods section.*
☒	☐	A description of all covariates tested
☐	☒	A description of any assumptions or corrections, such as tests of normality and adjustment for multiple comparisons
☒	☐	A full description of the statistical parameters including central tendency (e.g. means) or other basic estimates (e.g. regression coefficient) AND variation (e.g. standard deviation) or associated estimates of uncertainty (e.g. confidence intervals)
☒	☐	For null hypothesis testing, the test statistic (e.g. *F*, *t*, *r*) with confidence intervals, effect sizes, degrees of freedom and *P* value noted<br>*Give P values as exact values whenever suitable.*
☒	☐	For Bayesian analysis, information on the choice of priors and Markov chain Monte Carlo settings
☒	☐	For hierarchical and complex designs, identification of the appropriate level for tests and full reporting of outcomes
☒	☐	Estimates of effect sizes (e.g. Cohen's *d*, Pearson's *r*), indicating how they were calculated

Our web collection on statistics for biologists contains articles on many of the points above.

Software and code

Policy information about availability of computer code

Data collection	No software was used. All relevant codes are included in the Method section.
Data analysis	ImageJ, version 2.0.0-rc-68/1.52e; QuantStudio™ Real-Time PCR Software v1.3; trim_galore 0.4.0; Bowtie 0.12.9; mmseq-1.0.8; limma_3.30.4; Bowtie2; Homer; DESeq2 1.14.1; R version 3.3.2; Bioconductor version 3.4; topGo 2.26.0; hisat2; featureCounts ; MEME-suite;

For manuscripts utilizing custom algorithms or software that are central to the research but not yet described in published literature, software must be made available to editors/reviewers. We strongly encourage code deposition in a community repository (e.g. GitHub). See the Nature Research guidelines for submitting code & software for further information.

Data

Policy information about availability of data

All manuscripts must include a data availability statement. This statement should provide the following information, where applicable:

- Accession codes, unique identifiers, or web links for publicly available datasets
- A list of figures that have associated raw data
- A description of any restrictions on data availability

All RNA-seq and ChIP-seq data are available in the ArrayExpress database (www.ebi.ac.uk/arrayexpress) under the accession numbers E-MTAB-6995 and E-MTAB-6817. The uncropped western blots are provided in Source Data file. The source data underlying Figure Figs 1a-b, 1d, 1f, 1h-k, 2l, 3d, 3f, 3h-i, 3k, 4d-e, 5e-f and Supplementary Figs 1a-b, 1d, 1f-g, 1i, 1l, 2a, 2g, 3d-e, 3g, 5a, 5h-i are provided as Source Data file. All other relevant data supporting the key findings of this study are available within the article and its Supplementary Information files or from the corresponding author upon reasonable request.

Field-specific reporting

Please select the one below that is the best fit for your research. If you are not sure, read the appropriate sections before making your selection.

☒ Life sciences ☐ Behavioural & social sciences ☐ Ecological, evolutionary & environmental sciences

For a reference copy of the document with all sections, see nature.com/documents/nr-reporting-summary-flat.pdf

Life sciences study design

All studies must disclose on these points even when the disclosure is negative.

Sample size	RNA-seq, ChIP-seq and qPCR in Fig. S5A were performed with 2 biological replicates. All other data were performed with 3 biological replicates.
Data exclusions	No data were excluded.
Replication	biological replicates are samples that are prepared and measured independently throughout all the experimental procedures.
Randomization	No randomization was applied in this study.
Blinding	The investigators were not blinded to the samples.

Reporting for specific materials, systems and methods

We require information from authors about some types of materials, experimental systems and methods used in many studies. Here, indicate whether each material, system or method listed is relevant to your study. If you are not sure if a list item applies to your research, read the appropriate section before selecting a response.

Materials & experimental systems

n/a	Involved in the study	
☐	☒	Antibodies
☐	☒	Eukaryotic cell lines
☐	☐	Palaeontology
☐	☒	Animals and other organisms
☐	☒	Human research participants
☐	☐	Clinical data

Methods

n/a	Involved in the study	
☐	☒	ChIP-seq
☐	☐	Flow cytometry
☐	☐	MRI-based neuroimaging

Antibodies

Antibodies used	anti-HA (ab9110, Abcam and clone 16B12, Covance); anti-HA-agarose beads(A2095, Sigma); anti-KAP1 (ab22553, Abcam); IgG (12-371, Millipore); anti-Flag (F7425, Sigma); anti-Tubulin (T5168, Sigma)
Validation	Anti-HA (ab9110, Abcam) and anti-KAP1 (ab22553, Abcam) were validated for ChIP-seq. Anti-HA-agarose beads(A2095, Sigma) was validated for immunoprecipitation. Other commercial antibodies are validated for western blotting.

Eukaryotic cell lines

Policy information about cell lines

Cell line source(s)	3T3-L1 and HEK293T from ATCC; IBA cells (Pradhan et al., Scientific reports, 2017)
Authentication	No authentication was performed.
Mycoplasma contamination	The cells were not tested for mycoplasma contamination.
Commonly misidentified lines (See ICLAC register)	The study did not involve commonly misidentified lines.

Palaeontology

Specimen provenance	*Provide provenance information for specimens and describe permits that were obtained for the work (including the name of the issuing authority, the date of issue, and any identifying information).*

Specimen deposition	*Indicate where the specimens have been deposited to permit free access by other researchers.*
Dating methods	*If new dates are provided, describe how they were obtained (e.g. collection, storage, sample pretreatment and measurement), where they were obtained (i.e. lab name), the calibration program and the protocol for quality assurance OR state that no new dates are provided.*

☐ Tick this box to confirm that the raw and calibrated dates are available in the paper or in Supplementary Information.

Animals and other organisms

Policy information about studies involving animals; ARRIVE guidelines recommended for reporting animal research

Laboratory animals	Mouse, C57BL/6J and B6 Albinos.
Wild animals	This study did not involved wild animals.
Field-collected samples	This study did not involve samples collected from the field.
Ethics oversight	All experiments were approved by the ethics commission of the state veterinary office (60/2012. 43/2011).

Note that full information on the approval of the study protocol must also be provided in the manuscript.

Human research participants

Policy information about studies involving human research participants

Population characteristics	Obese patients with lipoaspirate surgery.
Recruitment	Fat tissues were collected from patients with lipoaspirate surgery.
Ethics oversight	Approved by the ethical commission of Canton Ticino (CE 2961 from 22.19.2015)

Note that full information on the approval of the study protocol must also be provided in the manuscript.

Clinical data

Policy information about clinical studies

All manuscripts should comply with the ICMJE guidelines for publication of clinical research and a completed CONSORT checklist must be included with all submissions.

Clinical trial registration	*Provide the trial registration number from ClinicalTrials.gov or an equivalent agency.*
Study protocol	*Note where the full trial protocol can be accessed OR if not available, explain why.*
Data collection	*Describe the settings and locales of data collection, noting the time periods of recruitment and data collection.*
Outcomes	*Describe how you pre-defined primary and secondary outcome measures and how you assessed these measures.*

ChIP-seq

Data deposition

☒ Confirm that both raw and final processed data have been deposited in a public database such as GEO.

☒ Confirm that you have deposited or provided access to graph files (e.g. BED files) for the called peaks.

Data access links<br>*May remain private before publication.*	http://www.ebi.ac.uk/arrayexpress/experiments/E-MTAB-6817
Files in database submission	Samples: HA_diff_rep1, HA_diff_rep2, HA_undiff_rep1; HA_undiff_rep2; IgG_diff_rep1; IgG_diff_rep2; IgG_undiff_rep1; IgG_undiff_rep2; KAP1m_diff_rep1; KAP1m_diff_rep2; KAP1m_undiff_rep1; KAP1m_undiff_rep2<br>For all samples, .fastq, .bam and .bed files are provided
Genome browser session<br>(e.g. UCSC)	Peak and aligned read files are available from Array Express and can be uploaded to a genome browser.

Methodology

Replicates	2 biological replicates each
Sequencing depth	HA_diff_rep1: 23701668; HA_diff_rep2: 25257753, HA_undiff_rep1: 23777833; HA_undiff_rep2: 24711875; IgG_diff_rep1: 23625921; IgG_diff_rep2: 22889298; IgG_undiff_rep1: 23065647; IgG_undiff_rep2: 14864931; KAP1m_diff_rep1:

25583016; KAP1m_diff_rep2: 32814613; KAP1m_undiff_rep1: 25325710; KAP1m_undiff_rep2: 26164471; alignment rate > 80% for all samples

Antibodies

Anti-HA (ab9110, Abcam) and anti-KAP1 (ab22553, Abcam) , IgG (12-371, Millipore)

Peak calling parameters

The ChIP-seq tags were aligned to the NCBI38/mm10 genome with Bowtie2 (Langmead and Salzberg, 2012) and the parameters "--very-sensitive -p 8". Duplicates were removed, as well as the reads with a mapping quality under 10. Regions showing significant ChIP enrichment (peaks) were determined with Homer (Heinz et al., 2010) ("- F 2 -L 3 -localSize 5000 -C 4 -fragLength 200 -minDist 500 -center") using matched IgG (for ZFP30-HA and KAP1) samples as controls. Read counts contained in the genomic intervals defined by ZFP30 or KAP1 binding, respectively, at day 0 to day 2 were compared using DESeq2 1.14.1 (Love et al. 2014) with padj ≤ 0.15, |FC| > 2.

Data quality

Reads with a low (under 10) mapping quality were removed. For peak calling, we used IgG as negative control. ZFP277-HA was additionally employed as a negative control based on its lack of localization to the nucleus, as previously described (Gubelmann et al., 2014): none of the called peaks overlapped ZFP277-HA enriched regions. Finally, read correlations inside peak regions were high for KAP1 and ZFP30-HA samples compared to IgG controls.

Software

Bowtie2 (Langmead and Salzberg, 2012)
Homer (Heinz et al., 2010)
DESeq2 1.14.1 (Love et al. 2014)

Flow Cytometry

Plots

Confirm that:

- [ ] The axis labels state the marker and fluorochrome used (e.g. CD4-FITC).
- [ ] The axis scales are clearly visible. Include numbers along axes only for bottom left plot of group (a 'group' is an analysis of identical markers).
- [ ] All plots are contour plots with outliers or pseudocolor plots.
- [ ] A numerical value for number of cells or percentage (with statistics) is provided.

Methodology

Sample preparation

Describe the sample preparation, detailing the biological source of the cells and any tissue processing steps used.

Instrument

Identify the instrument used for data collection, specifying make and model number.

Software

Describe the software used to collect and analyze the flow cytometry data. For custom code that has been deposited into a community repository, provide accession details.

Cell population abundance

Describe the abundance of the relevant cell populations within post-sort fractions, providing details on the purity of the samples and how it was determined.

Gating strategy

Describe the gating strategy used for all relevant experiments, specifying the preliminary FSC/SSC gates of the starting cell population, indicating where boundaries between "positive" and "negative" staining cell populations are defined.

- [ ] Tick this box to confirm that a figure exemplifying the gating strategy is provided in the Supplementary Information.

Magnetic resonance imaging

Experimental design

Design type

Indicate task or resting state; event-related or block design.

Design specifications

Specify the number of blocks, trials or experimental units per session and/or subject, and specify the length of each trial or block (if trials are blocked) and interval between trials.

Behavioral performance measures

State number and/or type of variables recorded (e.g. correct button press, response time) and what statistics were used to establish that the subjects were performing the task as expected (e.g. mean, range, and/or standard deviation across subjects).

Acquisition

Imaging type(s) — *Specify: functional, structural, diffusion, perfusion.*

Field strength — *Specify in Tesla*

Sequence & imaging parameters — *Specify the pulse sequence type (gradient echo, spin echo, etc.), imaging type (EPI, spiral, etc.), field of view, matrix size, slice thickness, orientation and TE/TR/flip angle.*

Area of acquisition — *State whether a whole brain scan was used OR define the area of acquisition, describing how the region was determined.*

Diffusion MRI ☐ Used ☐ Not used

Preprocessing

Preprocessing software — *Provide detail on software version and revision number and on specific parameters (model/functions, brain extraction, segmentation, smoothing kernel size, etc.).*

Normalization — *If data were normalized/standardized, describe the approach(es): specify linear or non-linear and define image types used for transformation OR indicate that data were not normalized and explain rationale for lack of normalization.*

Normalization template — *Describe the template used for normalization/transformation, specifying subject space or group standardized space (e.g. original Talairach, MNI305, ICBM152) OR indicate that the data were not normalized.*

Noise and artifact removal — *Describe your procedure(s) for artifact and structured noise removal, specifying motion parameters, tissue signals and physiological signals (heart rate, respiration).*

Volume censoring — *Define your software and/or method and criteria for volume censoring, and state the extent of such censoring.*

Statistical modeling & inference

Model type and settings — *Specify type (mass univariate, multivariate, RSA, predictive, etc.) and describe essential details of the model at the first and second levels (e.g. fixed, random or mixed effects; drift or auto-correlation).*

Effect(s) tested — *Define precise effect in terms of the task or stimulus conditions instead of psychological concepts and indicate whether ANOVA or factorial designs were used.*

Specify type of analysis: ☐ Whole brain ☐ ROI-based ☐ Both

Statistic type for inference
(See Eklund et al. 2016) — *Specify voxel-wise or cluster-wise and report all relevant parameters for cluster-wise methods.*

Correction — *Describe the type of correction and how it is obtained for multiple comparisons (e.g. FWE, FDR, permutation or Monte Carlo).*

Models & analysis

n/a	Involved in the study
☐	☐ Functional and/or effective connectivity
☐	☐ Graph analysis
☐	☐ Multivariate modeling or predictive analysis

Functional and/or effective connectivity — *Report the measures of dependence used and the model details (e.g. Pearson correlation, partial correlation, mutual information).*

Graph analysis — *Report the dependent variable and connectivity measure, specifying weighted graph or binarized graph, subject- or group-level, and the global and/or node summaries used (e.g. clustering coefficient, efficiency, etc.).*

Multivariate modeling and predictive analysis — *Specify independent variables, features extraction and dimension reduction, model, training and evaluation metrics.*
